# Supplementary material for: The Hippo pathway regulates density-dependent proliferation of iPSC-derived cardiac myocytes
Source: Sci Rep. 2021 Sep 7;11:17759. doi: 10.1038/s41598-021-97133-6 (PMC8423799; doi:10.1038/s41598-021-97133-6)
Supplement: Supplementary file 1 — Supplementary Figure S1. [file 41598_2021_97133_MOESM1_ESM.pdf]

Supplemental Material

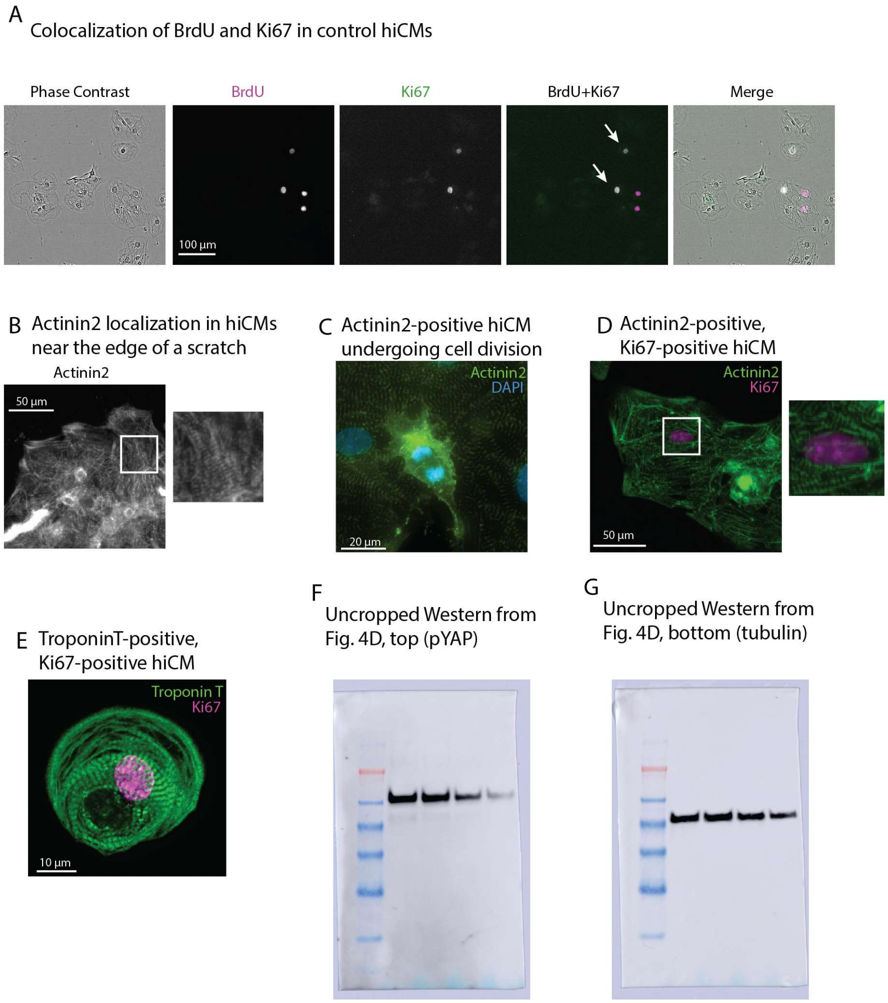

Figure S1

A) Colocalization of BrdU and Ki67 in control, sparsely-plated hiCMs. Phase contrast data shows that Ki67 and BrdU localize to nuclei in cardiac myocytes. B) Actinin2 localization in cardiac myocytes at the edge of a scratch, showing that cells at the edge of the scratch have sarcomeres. C) Actinin2 localization in a dividing cardiac myocyte, showing that dividing cells are actinin2-positive. D) Actinin2 and Ki67 localization in control cardiac myocytes, showing that Ki67-positive cells are actinin2-positive. E) TroponinT and Ki67 localization in control cardiac myocytes. F-G) Uncropped western blots from Figure 4. The ladder on the left of the blot is the SeeBlue Plus 2 standard from Invitrogen (Cat # LC5925).
